# Etude des anomalies du rythme cardiaque fœtal observées à l’examen cardiotocographique à Lubumbashi: cas suivis aux Cliniques Universitaires de Lubumbashi et à l’Hôpital Général du Cinquantenaire Karavia

**DOI:** 10.11604/pamj.2018.30.278.13365

**Published:** 2018-08-17

**Authors:** Joseph Chola Mwansa, Albert Mwembo Tambwe, Jules Ngwe Thaba, Arthur Munkana Ndoudule, Baudouin Yumba Museba, Thérèse Mowa Thabu, Prosper Kalenga Muenze

**Affiliations:** 1 Département de Gynécologie-Obstétrique de la Faculté de Médecine de l’Université de Lubumbashi, Republique Démocratique du Congo; 2 Service de Gynécologie-Obstétrique de l’Hôpital Général du Cinquantenaire Karavia, Lubumbashi, Republique Démocratique du Congo

**Keywords:** Cardiotocographe, souffrance fœtale aigue, facteurs associés, Lubumbashi, Cardiotocography, acute fetal distress, associated factors, Lubumbashi

## Abstract

L'usage du cardiotocographe (CTG) est très récent à Lubumbashi mais aucune étude approfondie n'est encore menée pour dégager son impact sur la morbi-mortalité périnatale. L'objectif de cette étude est de déterminer la fréquence des anomalies du rythme cardiaque fœtal (RCF), d'en rechercher les facteurs associés afin de proposer une prise en charge appropriée. Il s'agit d'une étude descriptive transversale réalisée durant 19 mois (de mars 2015 à décembre 2016) chez 411 parturientes. En présence des anomalies pathologiques du RCF, la sensibilité et la valeur prédictive positive de l'examen cardiotocographique, dans le dépistage de la souffrance fœtale aigue étaient respectivement de 82,95 et 45,35%. Les anomalies du RCF étaient retrouvées chez deux parturientes sur cinq. Les décélérations étaient les plus fréquentes de toutes les anomalies du RCF observées (50,8%) avec une prédominance remarquable des décélérations tardives (22,1% de toutes les anomalies). Les facteurs associés aux anomalies pathologiques du RCF étaient le travail prolongé (OR = 14,64, IC = 3,91-54,81), la chorioamniotite (OR = 14,56, IC = 3,83-55,34), l'anémie chronique maternelle (OR = 4,99, IC = 1,48-16,85), La primiparité (OR = 2,69, IC = 1,49-4,85), la prématurité (OR = 2,90, IC = 1,51-5,54) et la grossesse prolongée (OR = 3,22, IC = 1,38-7,52). Le Retard de Croissance Intrautérin et l'hypertension artérielle étaient associés particulièrement aux tracés plats et aux décélérations tardives (OR = 7,79, IC = 2,50-24,30 et OR = 2,74, IC = 1,31-5,72). Le CTG est un outil de dépistage de la souffrance fœtale aigue mais avec un taux élevé de faux positifs (55%); il convient de lui associer les autres moyens de dépistage de la souffrance fœtale aigue de seconde ligne en vue de réduire ce taux. Les facteurs associés aux anomalies pathologiques du RCF sont souvent à l'origine des causes de la souffrance fœtale aigue et exigent ainsi une analyse rigoureuse des tracés du CTG.

## Abstract (en)

Cardiotocography (CTG) has recently come into use in Lubumbashi but no thorough study has yet been conducted to identify its impact on perinatal morbi-mortality. This study aims to determine the frequency of fetal heart rate abnormalities (FHR)in order to identify the associated factors and to propose a suitable management. We conducted a cross-sectional, descriptive study of 411 women in labour over a period of 19 months (March 2015-December 2016). In patients with pathologic FHR abnormalities, sensitivity and positive predictive value of cardiotocography in the screening test for acute fetal distress were 82.95% and 45.35% respectively. FHR abnormalities were found in two women in labour out of five. Decelerations were the most frequent FHR abnormalities observed (50.8%) with a remarkable predominance of late decelerations (22.1% of all abnormalities). The factors associated with pathological FHR abnormalities were prolonged labor (OR = 14.64, CI = 3.91-54.81), chorioamnionitis (OR = 14.56, CI = 3.83-55.34), chronic maternal anemia (OR = 4.99, CI = 1.48-16.85), primiparity (OR = 2.69, CI = 1.49-4.85), prematurity (OR = 2.90, CI = 1.51-5.54) and prolonged pregnancy (OR = 3.22, CI = 1.38-7.52). Intrauterine growth retardation and arterial hypertension were mainly associated with flat lines and late decelerations (OR = 7.79, CI = 2.50-24.30 and OR=2.74, CI = 1.31-5.72). CTG is a screening tool for the identification of acute fetal distress but with high false-positive rate (55%); it should be associated with other second-line screening tests for acute fetal distress in order to reduce this rate. Factors associated with pathologic FHR abnormalities often cause acute fetal distress thus requiring a rigorous analysis of CTG traces.

## Introduction

L'usage du cardiotocographe (CTG) pour la surveillance continue du Rythme Cardiaque Fœtal (RCF) pendant le travail, est une technique mise en place depuis les années 1960 pour permettre aux Obstétriciens d'intervenir dans un délai raisonnable afin d'éviter les lésions fœtales dues à l'hypoxie ou l'asphyxie intrapartale [1]. L'analyse du tracé fourni par le CTG permet de retrouver dans certains cas des anomalies du RCF prédictives des états d'hypooxygénation fœtale et conduit l'équipe obstétricale à agir pour éviter l'asphyxie et la mortalité périnatale. Le versant tocogramme du CTG est toujours couplé au versant cardiographique pour une analyse optimale. La VPP peut atteindre 65% quand le CTG est utilisé seul et croit considérablement quand on lui associe les moyens de deuxième ligne comme la mesure du potentiel d'Hydrogène (PH) au scalp ou le dosage de la lactatémie, l'oxymétrie de pouls et l'analyse de l'électrocardiogramme (ECG) fœtal [1, 2]. En effet, le CTG a permis de réduire dans certains centres du monde l'acidose fœtale, l'encéphalopathie et le décès périnatals [3]. En Allemagne, le CTG a contribué à ramener dans les dix ans qui ont suivi son utilisation, la mortalité périnatale de 5,6 à 1,7 pour 1000 naissances [4]. En France, la mortalité périnatale est passée de 6 pour 1000 à la fin des années 1950 à 0.4 pour 1000 dans les années 1980 [5]. En Afrique subsaharienne, dans les centres où on n'utilise que l'auscultation intermittente dans la surveillance du travail, la mortalité périnatale avoisine 47 pour 1000 naissances alors qu'elle est de 4,8 pour 1000 naissances en Afrique du Sud où le CTG est régulièrement indiqué en cas de nécessité dans les hôpitaux modernes [6]. A Lubumbashi, la mortalité périnatale reste toujours élevée. Elle est de 27 pour 1000 naissances [7]. L'utilisation du CTG à Lubumbashi est toute récente mais aucune étude approfondie n'est encore menée pour dégager son impact réel sur la morbidité et la mortalité périnatale et les causes de celle-ci. Le présent travail a pour objectif de déterminer la fréquence des anomalies du RCF aux Cliniques Universitaires de Lubumbashi et à l'Hôpital Général du Cinquantenaire Karavia; d'en rechercher les facteurs associés afin de proposer une attitude pratique dans la surveillance fœtale pendant le travail en vue de contribuer à la réduction de la mortalité périnatale.

## Méthodes

Les Cliniques Universitaires de Lubumbashi et l'Hôpital Général du Cinquantenaire Karavia ont servi de lieu de récolte des données. En effet, ce sont les deux hôpitaux de la ville qui utilisent le CTG avec impression du tracé. Les autres centres qui en font usage sans imprimer les tracés n'ont pas été consultés ; le tracé étant la preuve forte de l'enregistrement. Il s'agit d'une étude descriptive transversale réalisée durant dix-neuf mois (soit de Mars 2015 à Décembre 2016) chez 411 parturientes et portant sur l'analyse des clichés instantanés des tracés enregistrés ainsi que sur l'analyse des relations entre certains facteurs associés. Les anomalies du RCF constituent l'événement étudié et les paramètres obstétricaux ont été analysés comme facteurs associés. Le score d'APGAR inférieur à 7 à la 5^ème^ minute a été considéré, dans cette étude, comme le gold standard définissant l'installation de la souffrance fœtale aigue. Ainsi, à partir des anomalies du rythme cardiaque fœtal, nous avons calculé la validité du CTG dans le dépistage de la souffrance fœtale aigue. Vu la diversité d'interprétations du tracé du RCF au cours du travail, l'étude a utilisé la classification du Collège National des Gynécologues et Obstétriciens Français de 2007 (CNGOF 2007) pour regrouper les anomalies du RCF et en faciliter l'analyse. Ainsi, les anomalies classées risque important et majeur d'acidose ont été dénommées « anomalies pathologiques du RCF »; les autres anomalies étaient dénommées « anomalies intermédiaires du RCF ». La taille de l'échantillon a été calculée en considérant que c'est la première étude dans notre environnement et que la proportion estimée des parturientes présentant au moins une anomalie cardiotocographique a été fixée à 50%. La taille minimale de l'échantillon étant de 384 parturientes, nous avons analysé pour l'ensemble de l'étude les tracés de 411 parturientes. Le seuil de signification, p, a été fixé à 0.05. Les tracés pris en compte sont ceux qui ont été enregistrés pendant au moins 30 minutes au cours de la phase active du travail et pour des parturientes dont l'âge de la grossesse était d'au moins 32 SA. Nous n'avons retenu qu'un tracé pour une parturiente. Les grossesses gémellaires ont été exclues de l'étude par manque d'un appareil adapté. Les parturientes sous des thérapeutiques altérant le RCF ont aussi été mises à l'écart. L'étude a été précédée par une phase de pré-test d'un mois pour optimaliser la récolte des données. L'analyse des données à l'aide des logiciels Epi Info et Excel a permis d'avoir les résultats ci-dessous.

## Résultats

**Figure 1:**
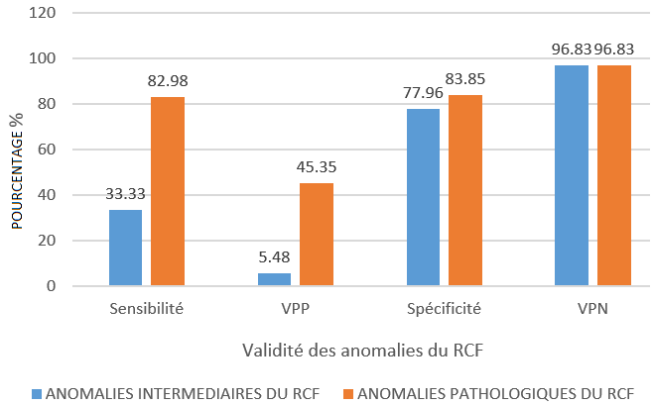
Sensibilité et vpp de l’examen cardiotocographique en présence des anomalies pathologiques et intermédiaires du RCF

**Validité de l'examen cardiotocographique:** Les fœtus avec RCF normal étaient au nombre de 252. Huit nouveau-nés avaient un score d'APGAR < 7 à la 5^ème^ minute. Nous avons eu 86 fœtus qui ont présenté des anomalies pathologiques; à la naissance, 39 nouveau-nés avaient un score d'APGAR < 7 à la 5^ème^ minute. Septante trois fœtus présentaient des anomalies intermédiaires; parmi eux, 4 nouveau-nés avaient un score d'APGAR < 7 à la 5^ème^ minute. En présence des anomalies pathologiques du RCF, la sensibilité, la VPP de l'examen cardiotocographique étaient respectivement de 82.98 et 45,35%. Ces valeurs étaient supérieures à celles observées en présence des anomalies intermédiaires qui étaient de 33,33 et 5,48%. Quant à la VPN, elle valait 96,83% (Figure 1).

### Anomalies du rythme cardiaque fœtal observées

**Fréquence globale des anomalies du RCF observées:** Dans cette étude, nous avons observé que 38.7% des foetus (soit 159 foetus) présentaient chacun au moins une anomalie du RCF.

**Figure 2:**
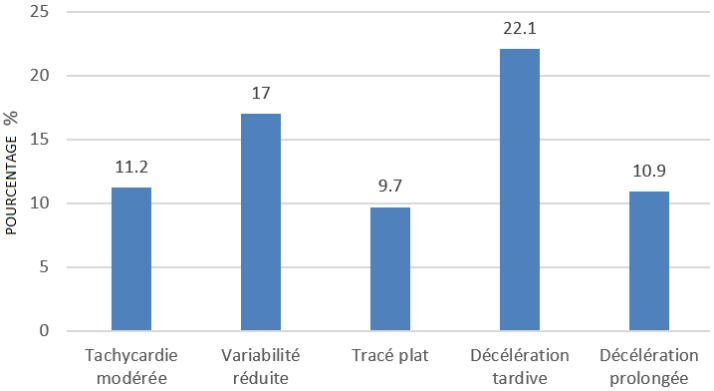
Principales anomalies du RCF observes

**Principales anomalies du RCF observées:** En analysant les anomalies du RCF observées, il se dégage de la Figure 2 que les cinq anomalies plus fréquentes étaient respectivement la décélération tardive (22,1%), la variabilité réduite (17%), la tachycardie modérée (11,2%), la décélération prolongée (10,9%) et le tracé plat (9,7%).

**Figure 3:**
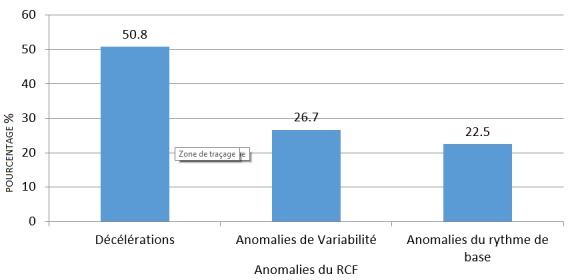
Répartition des anomalies du RCF selon qu’il s’agit d’une décélération, d’une anomalie de variabilité ou d’une anomalie du rythme de base

Répartition des anomalies du RCF selon qu'il s'agit d'une décélération, d'une anomalie de variabilité ou d'une anomalie du rythme de base: La Figure 3 montre que, de toutes les anomalies observées, les décélérations étaient de loin les plus fréquentes avec 50,8% de cas. Elles étaient suivies des anomalies affectant la variabilité du RCF qui représentaient 22,7%. Les anomalies du rythme de base ont représenté 22,5%.

**Associations entre les différentes anomalies du RCF:** Les anomalies du RCF étaient souvent associées les unes aux autres: Les bradycardies étaient associées aux anomalies de la variabilité (variabilité réduite et tracés plats) dans 74% des cas. Elles étaient accompagnées des décélérations dans 87% des cas et particulièrement des décélérations tardives dans 43,5% des cas. Les tachycardies étaient, quant à elles, associées à une variabilité réduite et aux décélérations tardives dans 20 et 48,6% des cas. Les tracés plats n'ont dans aucun cas présenté d'accélérations alors que 52% d'entre eux présentaient des décélérations tardives. Ces dernières ont par contre présenté des accélérations dans 19,3% des tracés. Enfin, les décélérations précoces s'accompagnaient d'accélérations dans plus de 59% de cas.

### Analyse des facteurs associés aux anomalies du RCF

**Facteurs associés aux anomalies pathologiques du rythme cardiaque fœtal:** Il se dégage du Tableau 1 que le risque de survenue des anomalies pathologiques du RCF étaient multiplié par 14,64 dans le travail prolongé, par 12,46 dans la chorioamniotite et par 4.99 dans l'anémie chronique. Ce risque était multiplié par 2,69 dans la primiparité, par 2,90 dans la prématurité, et par 3,22 dans la grossesse prolongée. L'âge maternel supérieur à 35 ans éloignait les fœtus du risque de présenter les anomalies pathologiques du RCF.

**Tableau 1:** Facteurs associés aux anomalies pathologiques du rythme cardiaque fœtal

Facteurs	OR	IC	p
**Age**			
Age < 20 ans	1,17	0,50-2,75	0,43
Age > 35ans	0,24	0,09-0,62	0,00
Age: 20-35 ans	1		
**Parité**			
Primipares (parité: 0)	2,69	1,49-4,85	0,00
Grande multipares (Parité> 7)	0,96	0,20-4,60	0,65
Multipares (Parité: 4-6)	1,0	0,45-2,47	0,53
Paucipares (parité: 2-3)	1		
**Age de la grossesse (SA)**			
Prématurité (32-36+6 jours)	2,90	1,51-5,54	0,00
Grossesse prolongée (> 42 SA)	3,22	1,38-7,52	0,00
Grossesse à terme	1		
**Anémie chronique**			
Anémie chronique	4,99	1,48-16,85	0,01
Absence d’anémie	1		
**Chorioamniotite**			
chorioamniotite	14,56	3,83-55,34	0,00
Fœtus sains	1		
Travail			
Travail prolongé	14,64	3,91-52,81	0,00
Dystocie mécanique	0,85	0,31-2,25	0,00
Dystocie dynamique	1,57	0,72-3,40	0,17
Eutocie	1		

**Facteurs associés aux tracés plats et aux décélérations tardives:** Le RCIU multipliait par 7.79 le risque de survenue des tracés plats. L'HTA multipliait par 2,74 le risque de survenue des décélérations tardives.

## Discussion

### La validité de la cardiotocographie

La sensibilité et VPP de l'examen cardiotocographique, en présence des anomalies pathologiques du RCF, étaient respectivement de 82.98 et 45.35%. La spécificité et la VPN étaient de 83,85 et 96,83 Larma et al [8] ont montré que la spécificité, VPP et la VPN étaient respectivement de 98,9, 50 et 88,6% lorsqu'on est en présence de bradycardie combinée aux variabilités réduites et à l'absence d'accélération. Holzman et al [9] ont trouvé que la VPP de la cardiotocographie était comprise entre 33 et 49% pour les décélérations tardives, les décélérations variables sévères et les tachycardies. En 2014, Tournemire [10] a montré que la sensibilité de l'enregistrement du RCF était de 88,8% lorsqu'on utilise la classification du CNGOF 2007 et la VPP est comprise entre 67,3 et 77,2% si le RCF est interprété par des experts. En établissant la Concordance entre la gazométrie au cordon et la classification du RCF selon le CNGOF, Dubourdeau et al [11] ont fixé en 2014 la sensibilité à 80% et la VPN à 96,9%.En 2016, Ana et Edwin [1] ont montré, en combinant toutes les anomalies, que la VPP de la cardiotocographie était de 30%, ce qui est nettement inférieur à nos résultats. Il est évident que le taux de faux positifs (FP) est assez grand pouvant aller de 22% à plus de 50%. C'est ce qui fait du CTG un outil de dépistage plutôt qu'un outil de diagnostic de la souffrance fœtale aigue [12]. La validité de la cardiotocographie telle que calculée à Lubumbashi ressemble bien à la validité calculée ailleurs surtout lorsque l'on tient compte essentiellement des anomalies graves du RCF.

### La fréquence des anomalies cardiotocographiques

Près de deux parturientes sur cinq (38,7%) ont présenté les anomalies du RCF. Parer et Ikeda [13] ont constaté qu'il y a près de 50% des tracés anormaux (incertains) au cours du travail. Ceci semble à première vue comme une fréquence très élevée; cependant toutes les anomalies du RCF ne sont pas prédictives des états d'acidose fœtale. Certaines sont banales sans signification (l'onde lambda, les décélérations indéfinies), d'autres sont transitoires et éphémères ou sont liées à des phénomènes physiologiques (le sommeil entraine une réduction de la variabilité), d'autres encore exigent qu'elles perdurent pour revêtir un caractère pathologique et que l'accouchement pourrait se produire avant même d'attribuer une signification à ces anomalies [14]. Quelques fois elles traduisent les malformations du cœur fœtal [15]. La réduction de la variabilité prise isolément n'est pas associée à un risque d'acidose. Les spikes, des décélérations indéfinies et l'onde lambda ne sont associés à aucun risque d'acidose fœtale. Les décélérations précoces prennent une signification particulière quand elles se prolongent au-delà d'une heure, leur amplitude dépasse 60 battements et leur nadire va en dessous de 80 battements [16]. Il n'y a qu'une portion d'anomalies du RCF qui détient la valeur prédictive d'acidose fœtale [17]. C'est d'ailleurs cela qui a amené beaucoup de sociétés savantes à établir des protocoles d'interprétation du RCF [18, 19].

Dans cette étude, les décélérations étaient les plus fréquentes de toutes les anomalies. Elles ont représenté la moitié des anomalies du RCF observées. Les décélérations variables sont les plus fréquemment rencontrées car elles représentent 30 à 40 % des tracés. En analysant les anomalies observées dans cette étude, il a été noté que les décélérations tardives étaient les plus fréquentes avec 22,1% d'anomalies. Ceci s'explique par les raisons ci-haut et que dans cette étude, l'enregistrement du RCF n'était pas systématique mais plutôt indiqué sur base de présomption de souffrance fœtale aigue. En plus, en analysant les décélérations variables, le caractère tardif a été le plus considéré car il augmente la valeur prédictive de l'acidose qui leur est attribuée [20, 21]. Les anomalies du RCF étaient associées les unes aux autres. Dans cette étude, les bradycardies et les tachycardies étaient associées aux variabilités réduites et aux décélérations. Les tracés plats ne présentaient pas d'accélérations et s'accompagnaient des décélérations. L'association d'anomalies du RCF augmente la sensibilité et prédiction du CTG dans le dépistage de l'acidose fœtale. La plupart d'anomalies, quand elles sont prises isolément, ne sont pas associées à une acidose fœtale. C'est le cas de la variabilité réduite ou la tachycardie [9]. Lorsque l'acidose métabolique s'est installée, les paramètres de régulation du RCF s'altèrent et cette altération conduit à des anomalies du RCF. L'hypoxémie active le système sympathique, d'où une tachycardie; l'hypoxie et l'asphyxie entrainent un dysfonctionnement des chémorécepteurs et des barorécepteurs avec une réduction progressive de la variabilité, ce qui conduit vers une variabilité réduite. En même temps, la réaction à une baisse brusque de la pression partielle en oxygène dévient lente par atteinte du système parasympathique; c'est cela qui explique la survenue de la composante tardive des décélérations. L'acidose crée un dysfonctionnement des cellules myocardiques, c'est l'apparition de la bradycardie sans récupération. C'est de cette manière que les anomalies se retrouvent combinées les unes aux autres [14].

### Les facteurs associés aux anomalies du RCF

Les facteurs associés aux anomalies pathologiques étaient: le travail prolongé, la chorioamniotite, l'anémie maternelle chronique, la primiparité, la prématurité et la grossesse prolongée. Le RCIU multipliait par 7.79 le risque d'avoir des tracés plats. L'HTA multipliait par 2.74 le risque d'avoir les décélérations tardives. Les différents facteurs décrits ici sont des situations cliniques à risque d'asphyxie intrapartale parce que chacun d'eux altère à un certain degré les échanges gazeux au niveau de la barrière placentaire. Ils se confondent aux étiologies de l'asphyxie fœtale aigues au cours du travail [22, 23]. Ces différents facteurs associés sont les mêmes que ceux décrits par Tournemire [10] comme facteurs de risque d'asphyxie et événements sentinelles hypoxiques. Le travail prolongé épuise les réserves fœtales et expose aux traumatismes obstétricaux fœtaux surtout en cas de rupture des membranes. En effet, les décélérations tardives seraient le reflet d'une diminution des réserves énergétiques fœtales et d'asphyxie. Les décélérations précoces témoignent la compression céphalique au cours de la descente. Par ailleurs les décélérations variables sont des indicateurs de compression funiculaire [24]. La chorioamniotite entraine la fièvre maternelle, ce qui conduit à une tachycardie maternelle et fœtale. L'augmentation du métabolisme de base fœtale explique en partie cette tachycardie. La consommation accrue d'oxygène au niveau tissulaire conduit au métabolisme anaérobie avec production d'acide lactique (acidose métabolique fœtale) qui crée plusieurs formes d'anomalies du RCF. La chorioamniotite accroit la morbidité fœtale et néonatale [25].

L'anémie chronique maternelle crée au niveau fœtal une hypoxémie qui retentit généralement sur la croissance staturo-pondérale (RCIU). Cette hypoxémie évolue lorsqu'elle se prolonge vers une hypoxie et une acidose métabolique fœtale. L'hypoxémie augmente progressivement la fréquence cardiaque de base et l'hypoxie entraine le dysfonctionnement du système de régulation (le système cardiaque autonome et central) qui altère les réponses adaptatives au déficit en oxygène; il en résulte une réduction des décharges sympathiques et parasympathiques avec disparition progressive de la variabilité et des accélérations du RCF. Ce dysfonctionnement entraine aussi des décharges parasympathiques lentes conduisant à des décélérations tardives avec une longue récupération. L'acidose métabolique, quand elle s'installe, induit une vasodilatation périphérique et une augmentation des résistances vasculaires au niveau des organes vitaux fœtaux. En conséquence, le métabolisme myocardique est profondément altéré, les systèmes de régulation ne fonctionnent plus, le débit cardiaque s'effondre suite à une chute de la fréquence cardiaque (bradycardie), sans récupération possible [14]. La préclampsie atteint souvent les primipares; et elle est un facteur de risque de souffrance fœtale aigue [24]. La prématurité, l'hypotrophie et la post maturité sont des situations incitant à une prudence accrue dans l'interprétation des anomalies du RCF. Les prématurés ou hypotrophes sont pourvus de réserves limitées pour faire face au stress du travail. Les capacités d'adaptation du fœtus aux différentes agressions hypoxémiques ou hypoxiques dépendent en grande partie de ses réserves, donc de sa trophicité et de son terme. La diminution des réserves les exposent à un grand risque d'asphyxie [5]. Les anomalies principales associées au RCIU sont la disparition des accélérations, le tracé est alors dit non-réactif, la diminution de la variabilité et les décélérations tardives [26, 27]. Dans cette étude, l'Hypertension Artérielle était particulièrement associée aux décélérations tardives. En effet, les décélérations tardives sont le reflet d'une diminution des réserves énergétiques. Cette condition est observée dans le RCIU et la prématurité qui peuvent être causés par l'Hypertension Artérielle. Dans la grossesse prolongée, la sénescence placentaire pourrait aboutir à une insuffisance des échanges materno-foetaux. Dans ce cas, l'adaptation normale du fœtus à l'hypoxie, principalement par redistribution du débit sanguin vers le cœur et le cerveau, peut-être déjà engagée, interdisant toute marge de sécurité [5]. Quant à l'âge supérieur à 35 ans, nous pensons qu'il pourrait être un facteur de confusion.

## Conclusion

La présente étude a montré que la sensibilité et la valeur prédictive positive du CTG dans le dépistage de la souffrance fœtale aigue étaient de 82.95 et 45.35% pour des anomalies graves du RCF. Par ailleurs, elle a indiqué que les anomalies du RCF étaient fréquentes au cours du travail (deux parturientes sur cinq) mais il n'y a qu'une portion de ces anomalies qui prédisent la souffrance fœtale aigue. Les différentes anomalies observées étaient associées les unes aux autres. Les facteurs associés aux anomalies pathologiques du RCF étaient le travail prolongé, la chorioamniotite, l'anémie chronique, la nulliparité, la prématurité, la grossesse prolongée, le retard de croissance intrautérin ainsi que l'Hypertension Artérielle. Ils sont souvent à l'origine des causes de l'asphyxie intrapartale et exigent ainsi une analyse rigoureuse des tracés du CTG.

### Etat des connaissances actuelles sur le sujet

- Le CTG est utilisé dans le monde dans la surveillance du travail.

### Contribution de notre étude à la connaissance

- Ce travail apporte les premières expériences de l'utilisation du CTG à Lubumbashi.

## Conflits d’intérêts

Les auteurs ne déclarent aucun conflit d'intérêt.

## Remerciements

Nous remercions tout le personnel de la maternité des Cliniques Universitaires de Lubumbashi et de l'Hôpital Général du Cinquantenaire Karavia.
